# Supplementary material for: Maximizing the reusability of gene expression data by predicting missing metadata
Source: PLoS Comput Biol. 2020 Nov 6;16(11):e1007450. doi: 10.1371/journal.pcbi.1007450 (PMC7673503; doi:10.1371/journal.pcbi.1007450)
Supplement: S1 Material — Supplementary text that include all the supplementary tables (Tables A-H). (DOCX) [file pcbi.1007450.s001.docx]

**S1 Material**

**Table A. F_1_-scores among different strategies in sequencing data.** RN denotes that the predictors were rank-normalized. SMOTE denotes that the samples were balanced by Synthetic Minority Over-Sampling Technique. Best number of genes selected by recursive feature elimination with cross validation.

|  | Race | | | ER^a^ | | |
| --- | --- | --- | --- | --- | --- | --- |
|  | RPM | RN | RN + SMOTE | RPM | RN | RN + SMOTE |
| LASSO | 0.709 | 0.800 | 0.829 | 0.695 | 0.743 | 0.747 |
| Random Forest | 0.546 | 0.575 | 0.746 | 0.826 | 0.838 | 0.824 |
| XGBoost | 0.816 | 0.793 | 0.854 | 0.839 | 0.800 | 0.809 |
| SVM | 0.529 | 0.891 | 0.863 | 0.618 | 0.776 | 0.732 |
|  | PR^b^ | | | HER2^c^ | | |
| LASSO | 0.667 | 0.759 | 0.734 | 0.500 | 0.522 | 0.573 |
| Random Forest | 0.757 | 0.775 | 0.766 | 0.572 | 0.561 | 0.582 |
| XGBoost | 0.776 | 0.752 | 0.763 | 0.593 | 0.592 | 0.616 |
| SVM | 0.625 | 0.727 | 0.714 | 0.480 | 0.545 | 0.535 |

a: Only patients with ER-positive (833) or ER-negative (243) were included.

b: Only patients with PR-positive (726) or PR-negative (347) were included.

c: Only patients with HER2-positive (173) or HER2-negative (581) were included.

**Table B. F_1_-scores among different settings using 10 selected genes in sequencing data.**

|  | Race | | | ER | | |
| --- | --- | --- | --- | --- | --- | --- |
|  | RPM | RN | RN + SMOTE | RPM | RN | RN + SMOTE |
| LASSO | 0.516 | 0.516 | 0.508 | 0.487 | 0.557 | 0.504 |
| Random Forest | 0.618 | 0.568 | 0.660 | 0.822 | 0.833 | 0.820 |
| XGBoost | 0.649 | 0.682 | 0.632 | 0.820 | 0.817 | 0.792 |
| SVM | 0.175 | 0.570 | 0.533 | 0.691 | 0.579 | 0.554 |
|  | PR | | | HER2 | | |
| LASSO | 0.509 | 0.454 | 0.542 | 0.000 | 0.158 | 0.440 |
| Random Forest | 0.785 | 0.785 | 0.788 | 0.599 | 0.583 | 0.554 |
| XGBoost | 0.765 | 0.746 | 0.726 | 0.586 | 0.535 | 0.551 |
| SVM | 0.594 | 0.745 | 0.766 | 0.536 | 0.560 | 0.519 |

**Table C. F_1_-scores among different settings using 25 selected genes in sequencing data.**

|  | Race | | | ER | | |
| --- | --- | --- | --- | --- | --- | --- |
|  | RPM | RN | RN + SMOTE | RPM | RN | RN + SMOTE |
| LASSO | 0.603 | 0.660 | 0.684 | 0.688 | 0.667 | 0.633 |
| Random Forest | 0.553 | 0.539 | 0.756 | 0.824 | 0.827 | 0.822 |
| XGBoost | 0.775 | 0.749 | 0.768 | 0.830 | 0.820 | 0.804 |
| SVM | 0.169 | 0.774 | 0.734 | 0.645 | 0.745 | 0.732 |
|  | PR | | | HER2 | | |
| LASSO | 0.628 | 0.640 | 0.733 | 0.360 | 0.398 | 0.462 |
| Random Forest | 0.770 | 0.761 | 0.764 | 0.588 | 0.577 | 0.563 |
| XGBoost | 0.791 | 0.761 | 0.740 | 0.593 | 0.552 | 0.548 |
| SVM | 0.539 | 0.739 | 0.726 | 0.470 | 0.538 | 0.527 |

**Table D. F_1_-scores among different strategies in microarray data.** RN denotes that the predictors were rank-normalized. SMOTE denotes that the samples were balanced by Synthetic Minority Over-Sampling Technique. Best number of genes selected by recursive feature elimination with cross validation.

|  | pCR | | | ER^a^ | | |
| --- | --- | --- | --- | --- | --- | --- |
|  | RPM | RN | RN + SMOTE | RPM | RN | RN + SMOTE |
| LASSO | 0.654 | 0.584 | 0.716 | 0.931 | 0.931 | 0.925 |
| Random Forest | 0.549 | 0.590 | 0.674 | 0.915 | 0.915 | 0.905 |
| XGBoost | 0.627 | 0.614 | 0.685 | 0.909 | 0.922 | 0.898 |
| SVM | 0.606 | 0.614 | 0.649 | 0.920 | 0.900 | 0.903 |
|  | PR^b^ | | | HER2^c^ | | |
| LASSO | 0.866 | 0.830 | 0.855 | 0.787 | 0.775 | 0.750 |
| Random Forest | 0.866 | 0.858 | 0.868 | 0.745 | 0.697 | 0.787 |
| XGBoost | 0.873 | 0.857 | 0.855 | 0.657 | 0.664 | 0.667 |
| SVM | 0.819 | 0.790 | 0.795 | 0.757 | 0.750 | 0.728 |

a: Only patients with ER-positive (516) or ER-negative (363) were included.

b: Only patients with PR-positive (465) or PR-negative (414) were included.

c: Only patients with HER2-positive (73) or HER2-negative (806) were included.

**Table E. F_1_-scores among different settings using 10 selected genes in microarray data.**

|  | pCR | | | ER | | |
| --- | --- | --- | --- | --- | --- | --- |
|  | RPM | RN | RN + SMOTE | RPM | RN | RN + SMOTE |
| LASSO | 0.095 | 0.130 | 0.395 | 0.890 | 0.711 | 0.708 |
| Random Forest | 0.539 | 0.518 | 0.578 | 0.901 | 0.903 | 0.907 |
| XGBoost | 0.480 | 0.508 | 0.586 | 0.900 | 0.881 | 0.889 |
| SVM | 0.293 | 0.297 | 0.450 | 0.871 | 0.836 | 0.875 |
|  | PR | | | HER2 | | |
| LASSO | 0.651 | 0.606 | 0.627 | 0.657 | 0.453 | 0.458 |
| Random Forest | 0.830 | 0.828 | 0.835 | 0.718 | 0.667 | 0.646 |
| XGBoost | 0.829 | 0.837 | 0.827 | 0.738 | 0.636 | 0.636 |
| SVM | 0.760 | 0.701 | 0.712 | 0.697 | 0.690 | 0.633 |

**Table F. F_1_-scores among different settings using 25 selected genes in microarray data.**

|  | pCR | | | ER | | |
| --- | --- | --- | --- | --- | --- | --- |
|  | RPM | RN | RN + SMOTE | RPM | RN | RN + SMOTE |
| LASSO | 0.372 | 0.367 | 0.464 | 0.874 | 0.814 | 0.840 |
| Random Forest | 0.578 | 0.563 | 0.600 | 0.914 | 0.914 | 0.919 |
| XGBoost | 0.616 | 0.584 | 0.584 | 0.918 | 0.904 | 0.897 |
| SVM | 0.432 | 0.300 | 0.523 | 0.880 | 0.873 | 0.875 |
|  | PR | | | HER2 | | |
| LASSO | 0.733 | 0.715 | 0.730 | 0.667 | 0.545 | 0.517 |
| Random Forest | 0.852 | 0.835 | 0.845 | 0.745 | 0.697 | 0.774 |
| XGBoost | 0.873 | 0.842 | 0.847 | 0.697 | 0.780 | 0.774 |
| SVM | 0.772 | 0.761 | 0.774 | 0.667 | 0.667 | 0.539 |

**Table G. F1-score using TCGA lung and brain tissue data.** The numbers are the average of 10-fold cross-validation. The models are trained on TCGA lung tissue samples and used to predict race and gender for TCGA brain tissue data.

|  | Race | | | Gender | | |
| --- | --- | --- | --- | --- | --- | --- |
|  | RPM | RN | RN + SMOTE | RPM | RN | RN + SMOTE |
| LASSO | 0.991 | 0.998 | 0.998 | 0.631 | 0.744 | 0.814 |
| Random Forest | 0.996 | 0.997 | 0.997 | 0.546 | 0.572 | 0.565 |
| XGBoost | 0.994 | 0.994 | 0.994 | 0.511 | 0.566 | 0.677 |
| SVM | 0.981 | 0.998 | 0.998 | 0.574 | 0.802 | 0.788 |

**Table H. F1-score using TCGA and GTEx data.** The numbers are the average of 10-fold cross-validation. The models are trained on TCGA data and used to predict tissue site for GTEx data.

|  | RPM | RN | RN + SMOTE |
| --- | --- | --- | --- |
| LASSO | 0.997 | 0.999 | 0.998 |
| Random Forest | 0.999 | 0.999 | 0.999 |
| XGBoost | 0.999 | 0.998 | 0.998 |
| SVM | 0.994 | 0.999 | 0.999 |
