# Supplementary material for: Maximizing the reusability of gene expression data by predicting missing metadata
Source: PLoS Comput Biol. 2020 Nov 6;16(11):e1007450. doi: 10.1371/journal.pcbi.1007450 (PMC7673503; doi:10.1371/journal.pcbi.1007450)
Supplement: Attachment — Submitted filename: PLOSCompBio_Review.pdf [file pcbi.1007450.s004.pdf]

Summary:

In this manuscript, the authors set out to utilize gene expression data (RNASeq; microarray) to predict breast cancer-specific phenotypes (ER, PR, and HER2 status), race (RNASeq), and treatment response (microarray). The authors generate predictions under a number of prediction scenarios, varying the (1) number of genes included in the model, (2) the normalization procedure, (3) the oversampling procedure utilized, and (4) the algorithm used. In this work, the authors propose a new metric for model assessment, PCAP - the proportion of cases accurately predicted given a specified accuracy threshold. Their results suggest that these binarized phenotypes can be accurately predicted from a subset of genes, that no one model performs consistently best, that PCAP is a good metric for future use, and suggest that Rank Normalization is a good procedure for use when working with multiple datasets.

Major Comments:

1. Sample Information
    - Important information about the samples is missing - Were these all primary tissue samples? Were some cell lines?
    - If cell lines, were these considered when estimating the amount of ER/HER2 information? Because, if they're cell lines, that metadata information should be available.
    - Why no estimate of what % of samples in GEO have missing PR in Introduction?
2. Differential Gene Expression Analysis
    - What question is being asked with the Differential gene expression analysis? It appears to be what genes are differentially expressed between AAs and CAs? However, your sample is unbalanced. Wouldn't it make more sense to do the analysis within race (given the aforementioned differences) using a breast cancer phenotype as your outcome variable? This would be more biologically meaningful.
    - Further, I may have missed them, but the approach/model/package used for DGEA analysis not included in the methods?
    - While I see in the analysis that there is not a difference between true and predicted percent of overlap (Figure 3)...they all look pretty close to random, which suggests that these results are not very meaningful. Was difference from random tested? I think if you pick a better comparison for your analysis, these results will be more informative/elucidating.
    - Figure 3 - would love this figure to show the underlying distribution of the data and not just the boxplot
3. Use by others
    - How would others go about using your procedure? Is the code available? Better yet, is there a python module/package that would allow others to repeat your work. I'd envision this having functionality to implement the many steps in your procedure (number of gene optimization, model implementation, PCAP calculation, etc.)

4. Figure 1
    - Is treatment response (phenotype used in microarray data) also available from TCGA? Would be helpful to see that same comparison in sequencing data if using in microarray (and have this be discussed in text).
    - For sequencing data, should “intermediate” and “equivocal” be included in this figure? These data were excluded from analysis and the phenotype binarized, so seems like these should not be there. If left, would like negative and indeterminate to have more distinct colors so readers can make out visually. Would also like a comment in text as to why the proportions for ER/PR/HER2 differ between these two sources of data.
5. Algorithm
    - Would love a discussion as to why these four ML algorithms were chosen, among all the algorithms out there.
    - I’m unclear as to how the number of “optimal set of genes” was chosen and why 10, 25, or another number were used. What was the metric for feature elimination? How would someone else go about determining the optimal number of genes?

Minor Comments:
- I suggest using the word “inferring” instead of “imputing” in the sentence “Second, modern approaches based on machine learning technology are now widely used for imputing...” to ensure readers are clear about the difference between imputation and machine learning-based approaches
- Would love the term “recovered” to be clarified/defined for readers. Is this word synonymous with infer? Same as predict? Would love the same language to be used throughout for clarity.
- How many other ethnicities were left out of the analysis? Including all ethnicities would give you the opportunity to test your methods on a variable that is not binary.
- Would like to see the number of samples that are breast cancer patients in sentence “In TCGA data, ER, PR and HER2 status were only available for breast cancer patients (N=??).
- Reference seems to be missing in Oversampling methods section for sentence ending in “which tend to give more accurate predictions”
- Discussion missing about what information is lost when using RN for normalization. Benefits are discussed, but loss of information limits downstream analyses; would love if these were mentioned
- Would like more discussion about inference after prediction - there is a limitation of using expression and predicted metadata for downstream analyses when prediction of metadata was generated *from* the same samples’ expression data.
- In tables, red color text is used to demarcate both the highest and lowest values, depending on the table. I suggest using one color when demonstrating highest performer (i.e. Table 1) and a different color when highlighting a low performer (i.e. Table 3)

- Discussion missing as to why HER2 performs worse relative to other breast cancer-related phenotypes.
- Would be hard for others to utilize your procedure given the variance among the results. Discussion should include how others should proceed given your results.
- General references to previous prediction from expression data work appear to be missing. May want to check out the following...which may inform this work (or maybe not!):
    - https://www.nature.com/articles/s41598-018-19635-0
    - https://academic.oup.com/nar/article/46/9/e54/4920847 / https://www.nature.com/articles/nbt.3838
    - https://academic.oup.com/bioinformatics/article/33/18/2914/3848915
