# Supplementary material for: Maximizing the reusability of gene expression data by predicting missing metadata
Source: PLoS Comput Biol. 2020 Nov 6;16(11):e1007450. doi: 10.1371/journal.pcbi.1007450 (PMC7673503; doi:10.1371/journal.pcbi.1007450)
Supplement: Attachment — Submitted filename: RevisionReport.docx [file pcbi.1007450.s005.docx]

**Revision Report**

We thank all the reviewers for their constructive comments and suggestions. We have made substantial revision and as a result the manuscript has been significantly improved. We have provided point by point answers to all reviewers’ concerns. The modified parts were colored as red in the revised manuscript. We first summarize the major new experiments we have done in this round of revision.

In the original manuscript, we have studied 5 different clinical variables using data from 5 types of cancers. In the revision, we have selected 42 variables from TCGA metadata and applied our framework on all applicable TCGA samples (totally 11284) and a subset of samples from GTEx project (Figure 3). The conclusions are much more solid and new insights were obtained. Specifically, we found that when using predicted metadata in downstream analyses, one should use the subset of data, which can be predicted accurately, instead of using all the predicted data (Figure 5). The optimal cutoff that defines PCAP threshold for selecting a subset of samples is likely between 55%-80% and 70% (PCAP_70_) may be a reasonable default value to strike the balance between accuracy and sample sizes (Figure 6). Figure 3, 5 and 6 are new figures produced in this revision. In addition, more tables were provided in Supplementary Materials.

**Reviewer #1:**

Major Comments :

*Sample Information*

1. *- Important information about the samples is missing - Were these all primary tissue samples? Were some cell lines?* If cell lines, were these considered when estimating the amount of ER/HER2 information? Because, if they’re cell lines, that metadata information should be available.

**Response: They are all primary tissue samples. No cell lines were used.**

2. - Why no estimate of what % of samples in GEO have missing PR in Introduction?

**Response: Reporting the % of missing samples for a couple of variables aimed to just give readers some concrete ideas on the scale of the missingness of important clinical variables. It is not important which variables we used to illustrate this point. If the reviewer insists, we will find this value and include it in the next revision.**

Differential Gene Expression Analysis

3. - What question is being asked with the Differential gene expression analysis? It

appears to be what genes are differentially expressed between AAs and CAs?

However, your sample is unbalanced. Wouldn’t it make more sense to do the

analysis within race (given the aforementioned differences) using a breast cancer

phenotype as your outcome variable? This would be more biologically

meaningful.

**Response: We want to show that we can use predicted metadata as the grouping information in differential gene expression analysis when original data has missing values. The comparison between predicted data and original data can be done between two groups defined by any of the variables we studied. We chose race instead of others because we have done quite some studies in the past on the racial disparity of breast cancer. In Figure 5 and 6, we showed the DGEA results for a substantially more variables (42 in total).**

**When doing differential gene expression analysis, it is fine to have unbalanced two groups. Just like in two sample t-test, the sizes of the two samples can be very different.**

4. - Further, I may have missed them, but the approach/model/package used for

DGEA analysis not included in the methods?

**Response: We are using two-sample t-tests after taking log2 transformation for the data because t-test is very robust. More commonly used methods for this task would be DESeq2, EdgeR or Limma+voom. However, since all the comparisons were done using the same method, the comparison is fair. Using other DGEA methods may produce higher numbers of differentially expressed genes, but the overall conclusions will be the same because results from two-sample t-test usually has substantial overlaps with those of other DGEA methods.**

5. - While I see in the analysis that there is not a difference between true and

predicted percent of overlap (Figure 3) they all look pretty close to random,

which suggests that these results are not very meaningful. Was difference from

random tested? I think if you pick a better comparison for your analysis, these

results will be more informative/elucidating.

**Response: We have provided the p-values to show that random is significantly worse than true or predicted, while predicted and true are not significantly different. We added this paragraph on page 8.**

Figure 4 and significance tests among the four groups (True, PCAP_95_, PCAP_90_, Random) showed that when computing the number of overlapping DEGs, there is no significant difference between using true race information and using predicted race (p-values between True and PCAP_95_ or PCAP_90_ are 0.28 and 0.54, respectively). There is no significant difference between using predicted race and using the true race of newly collected data, either (p-value between PCAP_90_ and CollectNew is 0.09). Randomly permuting the race will make the result significantly different (p-value between True and Random is smaller than 10^-38^). This indicates that datasets with metadata predicted using our method can be used reliably in the downstream analysis.

6. - Figure 3 - would love this figure to show the underlying distribution of the data

and not just the boxplot

**Response: We use a violin plot to show the distribution in the revised manuscript.**

Use by others

7. - How would others go about using your procedure? Is the code available? Better

yet, is there a python module/package that would allow others to repeat your

work. I’d envision this having functionality to implement the many steps in your

procedure (number of gene optimization, model implementation, PCAP

calculation, etc.)

**Response: example code is available at** [**https://github.com/dz16e/Reusability**](https://github.com/dz16e/Reusability)**.**

Figure 1

8. - Is treatment response (phenotype used in microarray data) also available from

TCGA? Would be helpful to see that same comparison in sequencing data if

using in microarray (and have this be discussed in text).

**Response: In TCGA, the majority of response data is missing. For those that are not missing, there are many different types of treatments, making the sample sizes for different treatments too small to perform meaningful analysis. We added more results in the revision, where we applied our framework to 42 variables in TCGA data.**

9. - For sequencing data, should “intermediate” and “equivocal” be included in this

figure? These data were excluded from analysis and the phenotype binarized, so

seems like these should not be there. If left, would like negative and

indeterminate to have more distinct colors so readers can make out visually.

Would also like a comment in text as to why the proportions for ER/PR/HER2

differ between these two sources of data.

**Response: In this study, we focused on demonstrating a framework for maximizing reusability of gene expression data and we have limited the machine learning problem to binary classifications. For ER/PR/HER2, we only used positive and negative samples.**

We do not know the reason for the difference in the proportions of ER/PR/HER2 between the two sources of data. One data source (GEO) is older than the other one (TCGA). It is possible that the two data sources have different populations of breast cancer patients or they used different experimental methods for measuring ER/PR/HER2.

Algorithm

10. - Would love a discussion as to why these four ML algorithms were chosen, among all the algorithms out there.

**Response: We chose these algorithms because they are most commonly used for diverse sets of problems. The performances of these algorithms are usually quite good.**

11. - I’m unclear as to how the number of “optimal set of genes” was chosen and why

10, 25, or another number were used. What was the metric for feature elimination? How would someone else go about determining the optimal number of genes?

**Response: Since we have more than 20,000 genes, we applied recursive feature selection to select a smaller number of genes while maintaining prediction power. The number of genes selected can either be determined by cross validation or specified by the user. The numbers of 10, 25 are based on our previous knowledge on building models using gene expression data. Other values can certainly be tried. In addition, we also tried to identify the optimal number of genes using cross validation based on one of the performance metrics, such as F1-score, AUROC, etc.**

Minor Comments :

12. - I suggest using the word “inferring” instead of “imputing” in the sentence “Second,

modern approaches based on machine learning technology are now widely used for

imputing...” to ensure readers are clear about the difference between imputation and

machine learning-based approaches

**Response: We have made the change. Thanks.**

13. - Would love the term “recovered” to be clarified/defined for readers. Is this word

synonymous with infer? Same as predict? Would love the same language to be used

throughout for clarity.

**Response: We have changed “recovered” to “predicted”.**

14. - How many other ethnicities were left out of the analysis? Including all ethnicities would

give you the opportunity to test your methods on a variable that is not binary.

**Response: In this study, our focus is not on biological discovery. That is why we simplified the problems to binary prediction so that the paper and the readers can focus more on the framework we are developing. It is quite straightforward to extend the framework to multi-class predictions.**

15. - Would like to see the number of samples that are breast cancer patients in sentence “In

TCGA data, ER, PR and HER2 status were only available for breast cancer patients (N=??).

**Response: There are 1246 samples.**

16. - Reference seems to be missing in Oversampling methods section for sentence ending in “which tend to give more accurate predictions”

**Response: We have added references for this sentence.**

17. - Discussion missing about what information is lost when using RN for normalization.

Benefits are discussed, but loss of information limits downstream analyses; would love if

these were mentioned

**Response: We only used rank normalized gene expression to perform prediction on missing metadata. In downstream analysis, we used the original gene expression data. Therefore, there is no loss of information. In terms of prediction accuracy, RN is better or comparable to alternative normalization methods that maintain the continuity of the data.**

18. - Would like more discussion about inference after prediction - there is a limitation of using

expression and predicted metadata for downstream analyses when prediction of

metadata was generated from the same samples’ expression data.

**Response: This is a good question. The short answer is it depends on the predictive models we built and the type of downstream analyses we will perform. The key is whether the model we built is biased for the particular downstream analysis. That is whether the mistakes the model makes are random or occur disproportionally in certain subspace of the whole population. We have discussed this issue in Discussion, which is copied below:**

There is a possibility that the subset of the data that can be predicted with high accuracy is systematically different in some way from the whole dataset. In such cases, the conclusion one can draw will be limited to only the subset of the data that can be predicted with high accuracy. In real clinical settings, for example biomarker discoveries, one can use the predictive model to stratify patient population and only apply the discoveries (i.e. identified diagnostic biomarkers) to that population.

19. - In tables, red color text is used to demarcate both the highest and lowest values,

depending on the table. I suggest using one color when demonstrating highest performer

(i.e. Table 1) and a different color when highlighting a low performer (i.e. Table 3)

**Response: We now use bold font for the highest values.**

20. - Discussion missing as to why HER2 performs worse relative to other breast

cancer-related phenotypes.

**Response: Some metadata variables are hard to predict using gene expression values. We have provided results on a much larger set of variables (totally 42 from TCGA) in this revision. Among the 42 variables in TCGA, the model performance on some variables are much worse than others, which indicates that those variables, by nature, cannot be predicted with high accuracy using gene expression.**

21. - Would be hard for others to utilize your procedure given the variance among the results.

Discussion should include how others should proceed given your results.

**Response:** **We added the following in the Discussion.**

From Figure 6, we can see that not all the metadata can be predicted accurately. When applying our method, the best practice would be doing training and validation on observations with no missing values, then predicting missing values using the trained models. If it appears that a variable can be predicted well based on the performance on validation data, then the framework can be used to predict the variable and use the predicted values for downstream analyses.

22. - General references to previous prediction from expression data work appear to be

missing. May want to check out the following...which may inform this work (or maybe

not!):

- https://www.nature.com/articles/s41598-018-19635-0

- https://academic.oup.com/nar/article/46/9/e54/4920847/

<https://www.nature.com/articles/nbt.3838>

- <https://academic.oup.com/bioinformatics/article/33/18/2914/3848915>

**Response: Thank you for pointing out these papers. We have added them in the references.**

**Reviewer #2:**

1. Although the paper is described as the prediction of missing data in public databases, in essence it is addressing a specific problem of whether specific sample attributes (variables), such as ethnicity, estrogen receptor (ER) status, progesterone receptor status, and similar can be predicted from gene expression data and to what extent.

**Response: This is a part of our study. The main findings of our study are: 1. When using the predicted metadata for downstream analysis, one should not use all of them. Instead one should use the subset of the data, which can be predicted accurately. In the revised manuscript, we added two figures to demonstrate this point (Figure 5 and Figure 6); 2. Because of 1, when developing machine learning models, one should not aim to optimize the overall accuracy. Instead, one should optimize PCAP; 3. We have developed an algorithm and a pipeline for optimizing PCAP; and 4. We showed that accurately predicted metadata can be reliably used in downstream analyses. In the revised manuscript, we have illustrated this with a much larger set of variables (42 totally compared to 5 in the original manuscript).**

2. The link to the public data is that if, for instance, a breast cancer gene expression data contains data from patients some of which are ER positive and some ER negative, but the sample status is not given, one can ask if this is possible to derive from the data itself? One can ask this question within a particular dataset and across datasets. In the first setting one would take a dataset where this variable (e.g., ER status) is included in the annotation, hide it, train a machine learning model and then use cross-validation to assess the accuracy of the model. In the second setting, one would train the model on one dataset, and then test on a different dataset. The second approach makes sense, the first really does not.

**Response: Actually, the first setting can also be quite useful in terms of missing value imputation. For a dataset with missing values, one can build a machine learning model using the same dataset to predict the missing data.**

**The second setting is also what we aimed to address. However, the data we have are from recount2, which are all normalized using the same method, making the data less heterogeneous. Note that recount2 contain independent datasets from different sources. We use it because it has rich metadata, which are necessary for demonstrating our framework. Our assumption is that we should build models using a set of data and then apply the models to other datasets, which were generated using the same platform and normalized using the same method. As more and more genomics data are becoming available, this assumption will be more likely satisfied. For any one variable, say ER status, we may need to build multiple models for multiple platforms.**

**In the revised manuscript, we built models using one cancer type in TCGA and applied it to another cancer type. We also built models using TCGA data and applied it to GTEx data. The performances of the models are satisfactory.**

3. One could also think of an approach where several datasets are merged, jointly normalised and then model is trained on the merged dataset, but I cannot imagine why one would do this, rather than the second – effectively meta-analysis approach.

**Response: In a recent paper of ours (MatchMixeR: A Cross-platform Normalization Method for Gene Expression Data Integration, Bioinformatics, 2020, PMID: 31904810), we have showed that jointly normalized data can be more powerful than using meta-analysis.**

**One could do meta-analysis instead of merging all the data. But when there are missing metadata, they still need to be imputed/inferred/predicted.**

4. Unfortunately, I was not able to understand from the paper, which of these approaches the authors take (possibly this can be deduced by studying the supplementary material, but I did not do this, nor should a reader). I think that if the authors are invited to resubmit the paper, they should clarify this.

**Response: I think we provided some answers to this in item 2 and 3 above. In addition, recount2 is a dataset that merged many different studies and normalized the data using the same normalization method so that the whole dataset can be analyzed together. However, many of the samples (there are more than 60,000) have important metadata missing. One can build models using recount2 data and then predict those missing metadata in recount2. This can be quite useful. For other sequencing datasets not in recount2, we can process the raw data and normalize them using the same procedure as recount2. Then we can use the models we built using recount2 data to predict the metadata of these datasets.**

5. In addition, I am worried that instead of using well accepted methods for assessing the accuracy of machine learning algorithms, the authors have designed their own methods. I did not fully understand the rationale of this.

**Response: Our new framework aims to maximize the reusability of gene expression data. It has different goals than machine learning and it makes sense to have a different objective to optimize. The key different is machine learning aims to optimize the accuracy for the whole dataset, while our framework aims to maximize the proportion of samples that can be predicted above certain accuracy level. PCAP is designed for this very purpose. We showed that this is important for increasing the quality of downstream analyses.**

6. Finally, one has to ask what is the utility of this? The authors spend significant space in the manuscript discussing comparisons of various ML methods but is this really interesting? It would be more interesting to choose a wider range of variables (e.g., sex, age bracket, tumour stage, etc) and compare how well each can be predicted (cross datasets). The problem of course would be that most variables are specific to particular diseases, or particular studies.

**Response: For the utility of this, we provided some answers in item 2 and 4 above. In the revised manuscript, we have added substantially more variables (42 totally). As a result, the conclusions are much more solid and we also gained more insight from the additional studies.**

7. The authors claim that their missing value imputation approach help in downstream analysis, particularly in differential gene expression. It is not surprising that it does (though I was not able to easily understand the author proof of this), but one could just as well use a latent variable approach (i.e., assume that there are latent variables in the dataset, which need to be factored out) for this problem, an approach which is well familiar to statistical geneticists. Computationally imputing missing values in the public databases would be rather dangerous, as this might easily lead to circularity in the downstream analysis by other authors.

**Response: This is a good question. The short answer is it depends on the predictive models we built and the type of downstream analyses we will perform. The key is whether the model we built is biased for the particular downstream analysis. That is whether the mistakes the model makes are random or occur disproportionally in certain subspace of the whole population. We have discussed this issue in Discussion, which is copied below:**

There is a possibility that the subset of the data that can be predicted with high accuracy is systematically different in some way from the whole dataset. In such cases, the conclusion one can draw will be limited to only the subset of the data that can be predicted with high accuracy. In real clinical settings, for example biomarker discoveries, one can use the predictive model to stratify patient population and only apply the discoveries (i.e. identified diagnostic biomarkers) to that population.

8. To summarise, I do not think that the paper is written clearly enough, I think the manuscript contains too much philosophy around, e.g., FAIR principles, which are not really relevant to the main line of the presented research, and in the current form it does not represent a clear advancement of science.

**Response: We have revised the manuscript to try to make it clearer. In addition, we have performed substantially amount of new experiments. We hope these new experiments and the new results have further clarified what we had aimed to deliver. We thank you for the questions, which have helped us think deeper about the study and improve it from there.**

**Reviewer #3:**

In this study, we develop a framework to predict missing metadata of gene expression datasets to maximize their reusability. We propose a new metric called Proportion of Cases Accurately Predicted (PCAP), which is optimized in our specifically-designed machine learning pipeline. The problem is extremely important and relevant of ensuring high quality metadata and with the increasing amount of data, methods are required to tackle it. The paper proposes a method to predict missing metadata using machine learning methods and show that their predictions can be used in downstream analysis. However, I have concerns about the main research question, methodology design and evaluation.

Here are my detailed comments:

1. - “However, the majority of public genomic data do not contain enough metadata, which severely limits their reusability.” Please provide evidence for this.

**Response: In Introduction we stated some missing rate for certain variables in GEO database. “**We did an analysis of available breast cancer gene expression data generated by platform GPL570 (Affymetrix Human Genome U133 Plus 2.0 Array) in the GEO database and found that there are 29,631 samples, of which ~85% do not have ER information, ~88% do not have HER2 information, and ~95% do not have race information.”

2. - “It may seem quite straightforward to infer missing metadata using gene expression profiles, given the abundance of previous research works.” Claims like these must be supported by evidence.

**Response: In our manuscript, we mentioned some previous work with citations: “It has been shown that gene expression is very informative and highly predictive of various clinical outcomes, such as the status of biomarkers [16-22], tumor types/status [23-26], the risks of recurrence [27, 28] and survival [28-31], therapeutic response [4, 32-35], or other metadata [36-40].”**

3. - “In this study, we investigate the above issues to infer missing metadata using multiple gene expression datasets with a wide variety of machine learning methods.” This is too broad and vague. Which machine learning methods? Why those methods? Why those specific datasets?

**Response: In our experimental setting, predicting missing metadata is essentially a classification problem, where machine learning is widely used. We choose the four methods (LASSO, SVM, Random Forest, and XGBoost) because those are most commonly used method for a diverse set of problems. We chose those specific datasets (TCGA, GTEx, ect) because they have large numbers of samples and metadata, the kind of properties important for our study.**

4. - Overall, the main research question/hypothesis is missing.

**Response: It is unknown that whether gene expression data can be used to accurately predict a diverse set of missing metadata and whether the predicted metadata can be used reliably for downstream analyses. We added that sentence to the revised manuscript. I am also copying some of the sentences we have in the revised manuscript: “**Genomics data are not useful if necessary metadata are not available. Unfortunately, a large amount of such important metadata is missing in public genomics datasets. In this study, we proposed a framework for maximizing the reusability of public gene expression data by predicting the missing metadata using machine learning methods.”

“We propose a new metric called Proportion of Cases Accurately Predicted (PCAP), which is optimized in our specifically-designed machine learning pipeline.”

“So far, we have been assuming that we would like the prediction to be at least above certain accuracy. Is this necessary? Should one use all the predicted data instead? To answer these questions, we looked at the performance of DGEA when using subsets of data with different levels of accuracy.”

“We found that when using predicted data to conduct other analyses, it is not optimal to use all the predicted data. Instead, one should only use the subset of data, which can be predicted accurately.”

“Using differential gene expression analysis as an example, we show that when missing variables are accurately predicted, the corresponding gene expression data can be reliably used in downstream analyses.”

5. - Another major concern is that there is no discussion of related work. What is the gap that the authors are trying to fill? There have been studies that have looked at methods to predict metadata:

-- Predicting biomedical metadata in CEDAR: A study of Gene Expression Omnibus (GEO) https://www.sciencedirect.com/science/article/pii/S1532046417301405

-- Predicting structured metadata from unstructured metadata https://academic.oup.com/database/article/doi/10.1093/database/baw080/2630448

-- massiR : a method for predicting the sex of samples in gene expression microarray datasets <https://academic.oup.com/bioinformatics/article/30/14/2084/2390865>

**Response: We added these citations in our revised manuscript. Thank you for pointing them out to us.**

**Traditional machine learning studies on predicting metadata aimed to maximize the accuracy of the prediction for all the data to be predicted. That is not our goal in this study. We proposed a new framework to maximize the proportion of samples, which can be predicted with high accuracy. This is important because from the new experiments we did in the revision, we found that when using predicted metadata for downstream analyses, one should not use all the predicted data. Instead, one should only use the subset of data which are predicted more accurately (Figure 5 and 6).**

**Previous studies never touched the topic of using predicted metadata for downstream analyses. In the revised manuscript, we applied our method to a wide range of variables (now totally 43 variables, 42 from TCGA and 1 from GEO).**

6. - This sentence “For each variable, the subset of samples with missing values was not considered in the analysis of the corresponding variable, since we cannot evaluate the performance of our method if we use these samples.” puzzled me because I thought that the aim of the project was to infer missing metadata.

**Response: We aim to develop a pipeline to infer missing metadata. But to evaluate the model performance, we need to have the non-missing original data, so that we can compare the predicted data with the ground truth. Observations with missing values cannot be used in training or validation.**

**Once our framework is validate using the data with no missing metadata, we can then use it for real data with missing metadata. In that case, the real accuracy will be unknown since nobody knows the true values of the missing data.**

7. - There doesn’t seem to be any novelty of the method that is used apart from an evaluation measure that they propose (PCAP - Proportion of Cases Accurately Predicted), which is also not evaluated. Only PCAP95 and PCAP90 seemed to have evaluated. How were these values determined? What is the threshold at which the accuracy decreases?

**Response: PCAP_95_ or PCAP_90_ were used to demonstrate that the framework works. In the revised manuscript, we have done a much larger study using many more variables and we actually found that we do not need to have a high accuracy cutoff for many variables. From Figure 6, one can see that for many variables the optimal cutoff will likely be between 55%-80%. From Figure 6, we can also see that this cutoff should also depend on the variable to be predicted. Figure 5 illustrate how the cutoff affects the performance of differential gene expression analysis.**

8. - In the “Normalization of gene expression” section, three normalization techniques are mentioned, whereas in the results sections only the effect of Rank Normalization is discussed. The other two methods’ results should also be discussed.

**Response: For the other two methods, one is for sequencing and the other is for microarray data. We compared RN with the alternative normalization method for both sequencing and array data (Figure 2 and 3).**

9. - I miss details on the “Model selection pipeline”, how does that work? There is no explanation of the pipeline.

**Response: This was discussed in page 4 and 5 under subsection Models.**

10. - The goal of predicting missing metadata is to use the corresponding gene expression data in other analyses.

- The example presented about the “differential gene expression analysis (DGEA) using predicted race” is a bit unclear. I would suggest to create a graphic to help understand it.

**Response: We used this example to show that predicted data can provide comparable result in downstream analysis as true data. We did additional experiments using more variables and showed the results in Figure 5 and 6. Hopefully, they help clarify what we aimed to do in Figure 4.**

10. - Additionally, it is insufficient to show that the predicted metadata can be useful in only one use case

**Response: We have done substantially more experiments using 42 variables and provided more results to solidify our conclusions (Figure 5 and Figure 6).**

11. - “recover a high percentage of samples with the desired accuracy” - please add more details for this

**Response: When a sample has an important metadata variable missing, such as ER status for a breast cancer sample, one cannot use the gene expression data of the sample to conduct studies that require ER status. When we predict the ER status using its gene expression data of this sample, we can now use the gene expression data of this sample. That is why we say “recover” a sample. Our goal is to recover as many samples as possible. However, when we predict the metadata, we also want to achieve high prediction accuracy. That is why we say “recover a high percentage of samples with the desired accuracy”.**

12. - Also, it is unclear whether the method can be generalized to other metadata

**Response: We applied our methods on 42 metadata variables in the TCGA dataset. The new results are shown in Figure 3, 5 and 6.**

13. - Additionally, the authors do not provide their code which makes it even more difficult to understand the implementation of the methodology.

**Response: example code is now available at** [**https://github.com/dz16e/Reusability**](https://github.com/dz16e/Reusability)**.**
