# Supplementary material for: Maximizing the reusability of gene expression data by predicting missing metadata
Source: PLoS Comput Biol. 2020 Nov 6;16(11):e1007450. doi: 10.1371/journal.pcbi.1007450 (PMC7673503; doi:10.1371/journal.pcbi.1007450)
Supplement: Attachment — Submitted filename: Review-PLOS-Comput.docx [file pcbi.1007450.s006.docx]

Review Comments on “Maximizing the Reusability of Gene Expression Data by Predicting Missing Metadata”

Pei-Yau Lung; Dongrui Zhong; Xiaodong Pang; Yan Li; Jinfeng Zhang

In this paper, the authors proposed to use machine learning methods to predict missing metadata for gene expression data in public databases. The idea is interesting and this will improve reusability of public genetic data, although no novel methodology is proposed. The prediction results are accurate. There are some major concerns as follows:

1. Only the binary metadata is predicted by using expression levels of selected genes as predictors. This formulation needs to clearly specified.
2. Each genetic sample has more than 20,000 genes, it is critical to select the significant genes (predictors) for the prediction model. Two-sample test may not be the best for predictor screening. The optimal predictor selection should be detailed.
3. Four metadata variables are predicted independently. Are these four metadata variables correlated? Can they be used to predict each other?
4. There are other missing data imputation approaches such as MICE. The standard missing data methods are not explored and compared with the proposed machine learning methods.
5. Only binary metadata variables are predicted. How to extend the methods to the categorical metadata variables?
6. The missing metadata problem is formulated into a standard supervised learning problem, i.e., use the labeled training dataset to build a prediction model to predict the unlabeled missing data. Different existing machine learning algorithms are evaluated under different conditions. The novelty of the proposed method is limited.
