# Supplementary material for: Maximizing the reusability of gene expression data by predicting missing metadata
Source: PLoS Comput Biol. 2020 Nov 6;16(11):e1007450. doi: 10.1371/journal.pcbi.1007450 (PMC7673503; doi:10.1371/journal.pcbi.1007450)
Supplement: Attachment — Submitted filename: Response to Reviewers.docx [file pcbi.1007450.s007.docx]

**Revision Report 2**

We thank all the reviewers again for their constructive comments and suggestions. We have made substantial revision and as a result the manuscript has been significantly improved. We have provided point by point answers to all reviewers’ concerns. The modified parts were colored as red in the revised manuscript.

Reviewer #1: *Generally, I would love exploration here and clarity in explanation/display of testing and am unsure how I would *use* this in the future if I found the results compelling. The two main conclusions seem to be:*

*1. Larger sample size not always the answer after metadata predictions used to supplement these points*

*2. PCAP a better metric*

*From the text and figures, I'm not convinced of these. I'm not saying they're untrue...I just struggle to draw these conclusions from the information provided. If these are not to be the takeaways, I'd revisit the text. If these are the main takeaways, I'd revisit the clarity of explanations provided and figures.*

**Response: These are essentially the main takeaways, which I will restate here: (1) when reusing existing gene expression data to predict a variable of interest and then do subsequent data analysis, it is not optimal to reuse all the available data. Instead, it is better to use the part of data for which we can predict the variable accurately. This is a new way of thinking when we reuse public gene expression data, which researchers have not explored before; (2) then the natural question is, how do we choose how much data to use? The answer is PCAP. PCAP is designed to select part of the data, which can be predicted with desired accuracy. There is no other metric one can use for this purpose since researchers have not investigated this problem before.**

Major:

- The manuscript has not become clearer in this round of revision. I'm not convinced that PCAP is a better approach and I'm even less clear about what predictions are being made, what cutoffs to use, how to determine ideal sample size after prediction, or what metrics to use to decide this. I think taking a hard look at the organization of these explanations and clarity in display of results would go a long way.

**Response: We provided the rational of our study using layman’s terms in Author Summary. We will give a very concrete example here to illustrate why we do this. For example, one wants to study gene expression difference between men and women in lung cancer using public data. In the public data, there are 100 lung cancer samples with gender information and 2000 lung cancer samples without gender information. There are three choices one can pick here:**

1. **Use only the 100 lung cancer samples to do the analysis.**
2. **This approach takes three steps: (1) use 100 lung cancer samples to build a predictive model for gender, which maximizes one of the traditional metrics designed for measuring the accuracy on the whole dataset, such as F1 score, AUC, etc.; (2) use the model to predict gender information for all the 2000 lung cancer samples; and (3) use all the 2000 lung cancer samples and the 100 lung cancer samples to do the differential gene expression analysis since now they all have gender information.**
3. **This approach also takes three steps: (1) use 100 lung cancer samples to build a predictive model for gender, which maximizes PCAP using our designed pipeline; (2) use the model to select a subset of 2000 lung cancer samples, for which gender can be predicted with high accuracy. Let’s say there are 1500 such samples; and (3) use the 1500 lung cancer samples and the 100 lung cancer samples to perform the subsequent differential gene expression analysis since now they all have gender information.**

**We have shown in our study that choice No. 3 is better than 1 and 2. Since this approach has not been investigated before, it is understandable it is a little hard to comprehend.**

- Figure 4 is very unclear to me. PCAP90 (the metric used in the manuscript) looks pretty darn close to random to me (on median); What about other metrics that you claim PCAP is better than? Why not included here?

**Response: We were showing the medians in the violin plots. We have changed to show both means and medians, which give clear difference between Random and the others.**

**We added the p-values of significance tests in the caption of Figure 4. From the p-values we can see that PCAP90 gives significantly different result from Random (PCAP90 is worse than PCAP95, which is what one would expect.). This is also discussed in “Results” section, “Using predicted information in statistical inference” subsection.**

**The idea of Figure 4 is to show that in the downstream analysis, the predicted information obtained by optimizing PCAP and the true information give similar results, while less accurate prediction (imitated by Random in Figure 4) gives much poorer result.**

- Figure 6 - without knowing what each point is, it's hard to know what variables you can predict with this method and if a different method would be better

**Response: A new version of Figure 6 is provided. We plot the index of the 42 metadata variables and provided a supplementary file “Figure6_variable_names.xlsx” to provide the mapping between the indices and the names.**

- Thanks for sharing the URL to the code in response to reviewers. Please include this in the manuscript directly.

**Response: We added the URL in the “Support information legend” section.**

Minor:

- First paragraph of introduction: "The availability of..." is missing a citation

**Response: Several citations were added.**

- Bottom of Page 3 (newly added material) - "It is unknown that whether" <- I think that is an additional word. If not, I'm not sure what this sentence is trying to say. Also, that sentence needs a citation or better wording. Also DGES is the initialism used here, but I think it's supposed to be DGEA

**Response: We have changed the wording of this sentence and moved it to an early part of Introduction. The modified sentence is: “However, as far as we know, no previous studies have shown that (1) gene expression data can be used to accurately predict a diverse set of missing metadata; and (2) the predicted metadata can be used reliably for downstream analyses.”**

**DEGS is a typo. We changed that to DEGA.**

- Page 7 - new content; "Our pipeline can be easily extended to multi-class predictions" <- either demonstrate this directly in manuscript or remove this statement

**Response: We have revised the sentence. One relatively simple way to extend binary classification to multi-class classification is to train multiple one-vs-rest models and summarize the predictions of these models to obtain the result for multi-class classification.**

- Top of pg 10 (new content) - unsure what the sentence "For variable," is conveying - have variables A,B,C, and D been introduced in text outside of figures yet?

**Response: We added the description of A, B, C and D in the main article.**

- Figure 1 colors - hard to compare between plots as negative and positive and the same color in (a) and (b)

**Response: We have updated Figure 1. Thanks for pointing this out.**

Reviewer #4:

*In this paper, the authors proposed to use machine learning methods to predict missing metadata for gene expression data in public databases. The idea is interesting and this will improve reusability of public genetic data, although no novel methodology is proposed. The prediction results are accurate.*

**Response: Thanks for the positive comments. We do consider the overall idea and framework to be novel.**

There are some major concerns as follows:

1. Only the binary metadata is predicted by using expression levels of selected genes as predictors. This formulation needs to clearly specified.

**Response: We emphasized the binary setting at the beginning of “Models” section.**

2. Each genetic sample has more than 20,000 genes, it is critical to select the significant genes (predictors) for the prediction model. Two-sample test may not be the best for predictor screening. The optimal predictor selection should be detailed.

**Response: We did not just use two-sample test to select features. We first use two-sample test to filter out some insignificant features, we then use recursive feature elimination (RFE) to select features from the remaining ones. This is discussed in the “Models” section. RFE is a commonly used feature selection method with good performance.**

3. Four metadata variables are predicted independently. Are these four metadata variables correlated? Can they be used to predict each other?

**Response: We treated metadata variables as responses. We showed the model performance on multiple metadata variables to demonstrate that our framework has wide range of applications. Those metadata variables may be correlated and added as predictors for each other.**

**Adding other metadata to predict a certain metadata is doable, but the usage of the resulting model could be limited if the other metadata variables are not available. In this paper, we choose to just use gene expressions as predictors for generality.**

4. There are other missing data imputation approaches such as MICE. The standard missing data methods are not explored and compared with the proposed machine learning methods.

**Response: When applying machine learning, we treat the non-missing instances as the training set, and predict the missing data using trained model. When doing imputation, we need to put together the non-missing and missing data (as a large data matrix with missing entries), then apply imputation methods such as MICE.**

**We ran a simple experiment to compare MICE and logistic regression. On a subset of microarray data used in the manuscript (879 observations, 1000 genes, 1 response), we randomly introduce 20% missing entries in the response, and use both MICE and logistic regression to infer the missing data. MICE imputed result only has an F1 score of 0.78, while logistic regression gives 0.90.**

5. Only binary metadata variables are predicted. How to extend the methods to the categorical metadata variables?

**Response: The proposed framework, including the PCAP metric, is model-agnostic. For example, in multi-class problems, one can build multiple binary one-vs-rest models and combine the results from these models for multi-class predictions. Also, the idea of optimizing PCAP is not limited to binary classification as long as we can define what “accurately predicted” (e.g., precision) is.**

6. The missing metadata problem is formulated into a standard supervised learning problem, i.e., use the labeled training dataset to build a prediction model to predict the unlabeled missing data. Different existing machine learning algorithms are evaluated under different conditions. The novelty of the proposed method is limited.

**It is true that predicting missing variables using machine learning methods is not novel. The novelties of our study are: (1) recognizing that one should not use all the available data when a missing variable need to be predicted. The better solution is to use a subset of data for which the missing variable can be accurately predicted. The approach will give better analysis performance in the downstream data analysis tasks. To the best of our knowledge, this has not been discussed in the past in the context of reusing public genomic data. Please also see the concrete example we gave early on for reviewer # 1; and (2) proposing a new metric, PCAP, that allows researchers to find out the subset of data that should be reused through optimizing that metric; and (3) developing a pipeline to optimize PCAP.**
